# Utilization of a Rehabilitation-Based Approach for Functional Movement Disorders: A Single-Center Experience

**DOI:** 10.7759/cureus.101339

**Published:** 2026-01-12

**Authors:** Catherine Schuster, Bianca Clyde, Kahir Jawad, Nicholas Yates, Aundrea Busse, Joshua Heath, Abbey Roach

**Affiliations:** 1 Pediatric Rehabilitation Medicine, University of Louisville School of Medicine, Louisville, USA; 2 Physical Medicine and Rehabilitation, University of Louisville School of Medicine, Louisville, USA; 3 Biostatistics, Norton Children's Research Institute, Louisville, USA; 4 Medical School, University of Louisville School of Medicine, Louisville, USA; 5 Psychology, University of Louisville Health (UofLHealth), Louisville, USA

**Keywords:** functional movement disorder, motor reprogramming, pediatrics, rehabilitation, rehabilitation outcomes

## Abstract

Background: Functional movement disorder (FMD) is a disorder of observable motor behavior, movement, or functional deficit that is incongruent with any underlying organic illness. This retrospective review aims to identify the benefit of inpatient rehabilitation for symptom burden reduction and examine possible temporal factors related to presentation and treatment timeframe that may impact symptom improvement.

Methods: A retrospective review was performed of patients admitted between January 1, 2015, to December 31, 2020, with a diagnosis of FMD under the specific program dedicated to rehabilitation management (Child and Adolescent Motor Reprogramming program). Information collected included demographics, type of symptom, time to diagnosis, time to admission, and longevity of symptom improvement at follow-up. WeeFIM® (Uniform Data System for Medical Rehabilitation, Buffalo, New York, United States) scores were also collected.

Results: During the study period, 38 patients were identified. All patients demonstrated improvement in symptom burden at discharge, and the majority noted ongoing improvements at follow-up. Odds ratios calculated for time to diagnosis and time to inpatient admission did not demonstrate statistical variance.

Conclusion: This study demonstrates that an inpatient rehabilitation approach can be beneficial for symptom burden improvement in functional movement disorders. Statistical analysis suggests that improvements can be achieved through this approach regardless of how long a patient has carried this diagnosis.

## Introduction

Functional movement disorder (FMD) or functional neurologic symptom disorder is a disorder in which a patient has an observable abnormal motor behavior, movement, and/or functional deficit that is not dangerous and is inconsistent with any underlying organic illness or disease. These abnormalities can manifest in many different ways, including gait disorders, tremors, tics, weakness, impaired coordination, abnormal postures or dystonic movements, visual disturbances, and other sensory issues. This diagnosis is classified under somatic symptom and related disorders, which also captures psychogenic nonepileptic spells or pseudoseizures. The common feature of these diagnoses is the prominence of somatic symptoms that results in significant distress and impairment [1]. The incidence of functional movement disorders is reported to be 4-12 per 100,000, but remains difficult to capture due to diagnostic challenges. Thenegannat et al. noted a prevalence of up to 6% of patients referred within one year to the neurology clinic [2]. Additionally, within pediatrics, up to 15% of new referrals to pediatric neurology receive an FMD diagnosis [3]. Similar to what has been noted within adult literature, symptoms in pediatric patients can be manifest in multiple different ways, with two of the most common being tremor and dystonia [4].

As stated in several previous studies, diagnosis of FMD can be difficult. LaFaver noted that many within the medical community have limited experience with FMD and that 32% of their survey responders indicated a lack of familiarity as a treatment barrier [5]. This limited familiarity adds to diagnostic challenges and may lead to associated disability and morbidity, such as “prolonged school absences and unnecessary surgical procedures in more than one-fifth of patients” [6]. However, diagnosis with appropriate terminology is an integral part of building the physician-patient rapport, establishing patient acceptance of the disease process (cessation of additional workup, imaging, or testing), and determining the trajectory of future treatment interventions. It has also been documented that patient acceptance of the diagnosis improves outcomes [7].

There is a growing preference for the term “functional” rather than its commonly associated “psychogenic” descriptor, thereby straying from using the diagnosis of conversion disorder. The Diagnostic and Statistical Manual of Mental Disorders, Fifth Edition (DSM-5) has removed “the need for the presence of a psychological stressor and exclusion of malingering” to make the FMD diagnosis [6]. Thus, “psychogenic” is a distracting descriptor with inappropriate implications regarding the physical disorders of our patients. Furthermore, the DSM-5 de-emphasizes “medically unexplained” symptoms, as these disorders can exist comorbidly with other medical diagnoses.

While FMD has been well-described in scientific literature, it is still a disorder with diagnostic and therapeutic challenges due to limited consensus within the medical field regarding etiology, terminology, and treatment strategies. LaFaver described changes in practices and opinions over the past decade in the treatment of FMD and concluded that while physicians are becoming more comfortable in diagnoses of FMD, variabilities in treatment approaches remain vast [5]. Some studies have achieved overall positive outcomes with approaches such as a monitored walking program [8], progressive physical therapy with verbal encouragement [9,10], cognitive behavioral therapy and psychotherapy [11], and pharmacologic intervention utilizing antidepressants [12]. A prospective study done by Herbert et al. noted the benefit of an inpatient rehabilitation approach initially, although they failed to find persistent long-term benefits in the 17 adult participants [13]. Presently, there is no consensus or established algorithm for the various methods of treatment as it relates to treatment strategies or management recommendations within this population, other than that both physical and psychological aspects must be addressed [14]. This is evidenced by Watson et al., who noted that there is high variability among treatment approaches and aftercare management within the United States [15]. 

The literature describing pediatric patients with FMD is even more sparse. Only recently did Kozlowska et al. demonstrate the benefit of a multisciplinary rehabilitation approach to improve outcomes in functional movement disorders with a focus on a mind-body connection and psychologically informed physiotherapy [14]. Despite this, many of the proposed treatment strategies are extrapolated from adult literature.

In this retrospective review of a single center's experience, the outcomes of inpatient rehabilitation under a motor reprogramming umbrella were examined with the aim of better understanding the role of a rehabilitation model for symptom burden reduction. This retrospective review aims to describe the outcomes of a cohort of pediatric patients diagnosed with functional movement disorder and detail how a multidisciplinary approach using motor reprogramming in an inpatient rehab facility setting can benefit pediatric patients suffering from FMD. The secondary aim is to determine the longevity of the success of the intervention regardless of the timing of diagnosis, as determined by subsequent follow-up appointments. Success within the secondary aim was primarily gauged by return to school and involvement in other typical pediatric pursuits and activities.

## Materials and methods

This was a retrospective study conducted at the University of Louisville. The study was approved by the Institutional Review Board of the University of Louisville (approval number: 20.0354). 

Study population

Patients evaluated between January 1, 2015, and December 31, 2020, were identified from electronic records by the diagnosis of FMD and admittance to the Child and Adolescent Motor Reprogramming program (CAMP). 

Study setting

The CAMP is a multidisciplinary treatment program within the rehabilitation institution for pediatric patients with FMD dedicated to restoring function and physical activities (attending school, participating in sports/activities, socializing with others, taking care of one’s body, etc). In the inpatient rehabilitation setting, patients are admitted and undergo frequent individualized therapy sessions with various disciplines, including physical therapy, occupational therapy, speech therapy, recreational therapy, and psychology. This program met all criteria for admission to an inpatient rehabilitation facility as outlined by the Centers for Medicare and Medicaid (CMS) [16]. The CAMP met all CMS inpatient rehab requirements with at least three hours of therapy per day, five to six days of the week. Additionally, within CAMP, psychologists met with each patient and family for daily one-hour sessions. Utilization of weekend hours encourages patients to gain independence by practicing exercises individually or with family; patients admitted to the program are given homework to accomplish outside of skilled therapy times. Parents/guardians play a key role as the support foundation. The amount of parent/guardian participation is dictated by the multidisciplinary team and varies based on the patient’s needs, and may include routine parent team conferences to discuss symptoms and symptom response, as well as independent exercise programs. A psychologist helps each patient develop an activity or coping plan through cognitive-behavioral therapy, relaxation training, and behavioral activation to ensure that each patient is prepared to manage his or her own symptoms at home. A physiatrist leads the multidisciplinary team and evaluates/manages medical needs daily. Since the diagnostic work-up is already complete, additional imaging, tests, or even medication adjustments are rarely needed. Insurance approval was obtained for every pediatric patient admitted to the program. 

Data collection

Demographics, type of functional movement or predominant symptom, WeeFIM® (Uniform Data System for Medical Rehabilitation, Buffalo, New York, United States) scores on admission and discharge [17], return to school status, and symptom burden at follow-up were recorded and analyzed. WeeFIM is an 18-point standardized assessment tool that measures a patient's functional independence in daily activities, measuring self-care, mobility, and cognition. Additionally, information regarding time to diagnosis and time to admission to inpatient rehabilitation was also collected.

Data analysis

Initial comparisons were done looking at time from reported symptom onset to diagnosis, which was generally given by a pediatric neurologist, and how this impacted return to school, symptom persistence at follow-up appointment, and continuance of therapies after discharge from an inpatient rehabilitation facility. Severity of symptom was not specifically captured; by nature of inpatient rehabilitation admission, the functional movement was significant enough to impact each patient's function. Patients were grouped into groups of greater or less than 30 days in time from diagnosis to CAMP admission in an effort to determine if outcomes were demonstrably better with faster time to CAMP admission. Odds ratios (ORs) were calculated comparing these two groups and how these differed in measured improvement outcomes. Patients were grouped into time to diagnosis of less than 7 to 180 days, and greater than 180 days. In the patient population, three categories of outcomes were measured: return to school, symptom persistence at follow-up appointment, and continuance of therapies after discharge from the inpatient rehabilitation facility. These three outcomes were scored as yes, no, or unknown.

Statistical analysis

The potential predictors used in the univariable analysis are time to diagnosis and time from diagnosis to CAMP categories. Multinomial logistic regression was performed to identify potential predictors for return to school, ongoing symptom persistence at follow-up appointments, and continuance of therapies after discharge from the inpatient rehabilitation facility. Polytomous logistic regression is used as the categories of the outcome variable have more than two categories and they do not have any natural order. ORs are reported with their 95% confidence intervals (CI) and two-sided p-values. A two-sided p-value < 0.05 was considered statistically significant. Statistical analysis was conducted using SAS software version 9.4 (SAS Institute Inc., Cary, North Carolina, United States).

## Results

A total of 38 participants were included in the study. Of these, 26 were female, 11 were male, and one was a transgender male participant. The average age of participants at the beginning of the study was 15 years, with the average age of symptom onset being 14. A total of 17 participants were admitted from home, 20 were admitted from an acute care children’s hospital, and one was admitted from an outside hospital. All patients underwent intensive inpatient rehabilitation with at least three hours of therapies per day in addition to supportive psychological services and integration with child life as appropriate (Tables 1, 2). The average admission WeeFIM score was 92.2, with an average discharge WeeFIM of 105.5. All patients were scheduled for outpatient follow-up, and of those who attended their scheduled appointments, half noted ongoing symptom improvement (Table 3).

**Table 1:** Patient demographics (N=38) FDI: functional disability index; IQR: interquartile range; LOS: length of stay; CAMP: Child and Adolescent Motor Reprogramming program

Characteristic	Frequency (Percentage)
Sex	
Female	26 (68.4%)
Transgender	1 (2.6%)
Male	11 (29.0%)
Location Admitted From	
Home	17 (44.7%)
Acute care children’s hospital	20 (52.6%)
Outside hospital	1 (2.6%)
Continued Therapies	
None	11 (29.0%)
Psychology support	21 (55.3%)
Other (modified)	6 (15.8%)
Persistence of symptoms at follow-up	
No	9 (23.7%)
Unknown	19 (50.0%)
Yes	10 (26.3%)
Return to School	
Yes	29 (76.3%)
No	5 (13.2%)
Other	4 (10.5%)
Residence from Rehabilitation Facility (miles)	
≤ 20	16 (42.1%)
> 20	22 (57.9%)
Time To Diagnosis (days)	
≤ 7	7 (18.4%)
7-180	17 (44.7%)
> 180	14 (36.8%)
Time From Diagnosis to CAMP (days)	
≤ 30	30 (79.0%)
> 30	8 (21.1%)

**Table 2:** Patient characteristics as median (IQR) FDI: functional disability index; IQR: interquartile range; LOS: length of stay; CAMP: Child and Adolescent Motor Reprogramming program

Parameters	Median (IQR)
Age at Admission (years)	15.0 (14-16)
Age of Onset (years)	14.0 (12-15)
Residence from Rehabilitation Facility (miles)	58.5 (0-165)
LOS (days)	10.0 (9-11)
FDI at admission	20.0 (17.0-34)
FDI at discharge	8.5 (1-14)
Time to Diagnosis (days)	105 (30-540)
Time from Diagnosis to CAMP (days)	7 (5-30)

**Table 3:** WeeFIM® score at different time periods SD: standard deviation

Time	WeeFIM score, mean±SD
Admission	92.2±8.6
Discharge	105.5±6.0
Improvement	13.3±7.4

When looking at return to school as a marker for age-appropriate reintegration, for each one-day increase in time from diagnosis to CAMP, the odds of returning to school were 0.1% less than not returning to school; however, the p-value was not significant. For every one-day increase in time from diagnosis to CAMP, the odds of return to school in a modified fashion (other) were 0.0% less than not returning to school. In looking at time to diagnosis, for each one-day increase, the odds of returning to school were 0.1% less, and the odds of returning to school in a modified fashion were 0.3% less when compared to not returning to school (Table 4).

**Table 4:** Return to school as multinomial logistic regression analyses showing the association between predictors (time from diagnosis and time to diagnosis)

Duration	Yes vs No, OR (95% CI)	Other vs No, OR (95% CI)
Time from Diagnosis (days)	0.994 (0.985–1.003)	1.000 (0.990–1.011)
Time to Diagnosis (days)	0.999 (0.997–1.000)	0.997 (0.993–1.001)

For ongoing symptom persistence at outpatient clinic follow-up, the odds of having continued symptoms were 0.1% less for each additional day in time from diagnosis to CAMP, while the odds of having unknown symptoms due to lack of follow-up were 0.1% less when compared to having no reported symptoms at follow-up. When analyzing time to diagnosis, for each one-day increase in time to diagnosis, the odds of having continued symptoms were 0.1% less, while the odds of having unknown symptoms due to lack of follow-up were 0.0% when compared to having no reported symptoms at follow-up (Table 5).

**Table 5:** Persistence of symptoms at followup as multinomial logistic regression analyses showing the association between predictors (time from diagnosis and time to diagnosis)

Duration	Unknown vs No, OR (95% CI)	Yes vs No, OR (95% CI)
Time from Diagnosis (days)	0.999 (0.990–1.008)	0.999 (0.990–1.009)
Time to Diagnosis (days)	1.000 (0.999–1.001)	0.999 (0.997–1.001)

When addressing the need for ongoing therapies, meaning either physical, occupational or speech compared to psychological, after discharge from the inpatient rehabilitation program, for every pne-day increase in time from diagnosis to CAMP, the odds of having no continued therapy were 1.007 times higher than having psychological support, and the odds of having other continued therapies were 12.3% less than having psychological support. For every one-day increase in time to diagnosis, the odds of having no continued therapy were 1.001 times higher than having psychology support alone, while the odds of having other continued therapies were1.001 times higher than having psychology support alone (Table 6).

**Table 6:** Continued therapies as multinomial logistic regression analyses showing the association between predictors (time from diagnosis and time to diagnosis)

Duration	None vs Psychology support, OR (95% CI)	Other (modified) vs Psychology support, OR (95% CI)
Time from Diagnosis (days)	1.007 (0.998–1.016)	0.877 (0.721–1.067)
Time to Diagnosis (days)	1.001 (0.999–1.002)	1.001 (0.999–1.002)

In this way, the calculated ORs demonstrated no significant effect on time to diagnosis or time to treatment. All patients included in the study reported symptom burden improvement at the time of CAMP discharge, regardless of how long they carried a diagnosis of FMD or how long before admission to inpatient rehab. Information regarding ongoing symptom improvement was limited due to a lack of follow-up, but those who could be tracked continued to report ongoing improvements. 

## Discussion

Currently, there is no consensus regarding treatment approaches for FMD. This single-center study shows how a multi-disciplinary approach focused on motor reprogramming in the inpatient rehabilitation setting can be effective in improving functional outcomes in pediatric patients diagnosed with FMD, as evident by the average overall WeeFIM score improvement of 13.3 (SD 7.4) and improvement in the Functional Disability Index. An improvement in WeeFIM demonstrates that functional gains were made through this rehabilitation approach. There is no consensus for improvement in WeeFIM across inpatient rehabilitation in functional movement disorders. 

The primary goal of this study was to determine the benefit of an inpatient rehabilitation model on symptom burden improvements. The secondary goal was to address whether the time to diagnosis of FMD and the time from diagnosis to our inpatient rehabilitation program impacted patient recovery. All patients had symptom improvement documented at the time of discharge from the inpatient rehabilitation setting. Additionally, all had a plan for returning to school (as appropriate) at the time of discharge. In a limited follow-up, ongoing symptom improvement was noted. The OR calculated for all investigated patient outcomes (return to school, symptom persistence, and ongoing therapy need) had confidence intervals that included 1, signifying that neither time to diagnosis nor time from diagnosis to CAMP had a significant impact on patient recovery. All patients had similar return to school rates, symptom persistence rates, and outpatient therapy continuance rates, regardless of time from diagnosis to treatment or time to diagnosis. This means that regardless of the time to FMD diagnosis or the time from diagnosis to CAMP, patients were able to achieve comparable outcomes. Thus, CAMP could be used as a viable interventional option for any FMD patient, irrespective of the time taken to receive an FMD diagnosis or the time from diagnosis to an acute inpatient rehabilitation setting. After discharge from CAMP, 85% of the patients noted little to no pervasive, ongoing FMD symptoms and had returned to school full-time by the time of outpatient follow-up evaluation. Only half of the patients returned for their routine scheduled follow-up appointment, with nine of the 19 reporting ongoing symptom improvement. Of the 19 that did not return to follow-up, it could be inferred that families did require ongoing rehabilitation involvement due to ongoing improvements resulting on lack of follow-up. Distance from the inpatient rehabilitation facility was measured as a secondary outcome, and it was found that there was no association or correlation in outcomes with populations of living less than or greater than 20 miles away from the treatment center.

Describing FMD as a disconnect between central, neurologic, and musculoskeletal communication has been successful and is the approach used when explaining our patients’ observed phenomena. Our retrospective study showed that a motor programming approach, like the one utilized through the CAMP, is a viable treatment option for many pediatric patients diagnosed with FMD and should be considered as a treatment strategy within inpatient rehabilitation facilities.

However, this motor reprogramming treatment approach may not be suitable for all FMD patients. As Czarnecki et al. reported, in patients with episodic movement disorders with long quiescent intervals, motor reprogramming cannot effectively be utilized during times of neurologic normality; patients with severe pain may have difficulty focusing and engaging in motor rehabilitation, and patients with many other somatic symptoms (somatization disorder) may be too distracted by their other symptoms to benefit [9].

Another drawback of this treatment form is the burden of insurance approval and the initial up-front cost. Not all insurance policies will cover acute inpatient rehabilitation, so participation in the CAMP is limited to those with insurance policy coverage or with the financial ability to pay for services out-of-pocket. All patients included in this study met the criteria for inpatient rehabilitation. In addition to financial costs for multi-disciplinary services and coordination, there are also patient/family costs, such as school days missed, parent/guardian workdays missed, and childcare costs for other children, which may act as a barrier for patients to enroll in the program. These financial and social barriers may limit patients' ability to participate in acute inpatient rehabilitation.

Limitations of the study

A limitation of this study was the use of retrospective analysis, which is inherently limited. A prospective study with a control arm would likely produce more significant results and provide additional evidence of the validity of the multidisciplinary team approach to motor reprogramming in the inpatient rehabilitation setting. Insurance barriers and the requirement to meet inpatient rehabilitation criteria also serve as a limiting factor in understanding the implications of who may benefit from this type of program, as not all patients have access to insurance coverage in general or specifically for inpatient rehabilitation; thus, some patients who may benefit from an inpatient rehabilitation approach may not have been captured. This study is also limited in that it represents a small sample of a single center, which is not necessarily generalizable nationwide. Additionally, characteristics and presentations of FMDs are variable, and not all symptoms were represented in our small sample.

## Conclusions

This retrospective study suggests that a rehabilitation approach to the treatment of FMDs can be beneficial for symptom improvement. Additionally, it showed that regardless of the time to diagnosis or rehabilitation treatment, improvements are still possible. More studies are needed to better determine characteristics of the patient population that would be amenable to positive outcomes, as well as the identification of ongoing treatment strategies to support symptom improvement. A larger cohort size would improve the generalization of the information. Future studies are needed with a control group to understand the specific role of inpatient rehabilitation. 
